# Mental Health Among Parents of Children Aged <18 Years and Unpaid Caregivers of Adults During the COVID-19 Pandemic — United States, December 2020 and February–⁠March 2021

**DOI:** 10.15585/mmwr.mm7024a3

**Published:** 2021-06-18

**Authors:** Mark É. Czeisler, Elizabeth A. Rohan, Stephanie Melillo, Jennifer L. Matjasko, Lara DePadilla, Chirag G. Patel, Matthew D. Weaver, Alexandra Drane, Sarah S. Winnay, Emily R. Capodilupo, Rebecca Robbins, Joshua F. Wiley, Elise R. Facer-Childs, Laura K. Barger, Charles A. Czeisler, Mark E. Howard, Shantha M.W. Rajaratnam

**Affiliations:** ^1^Turner Institute for Brain and Mental Health and School of Psychological Sciences, Monash University, Melbourne, Australia; ^2^Austin Health, Melbourne, Australia; ^3^Brigham & Women’s Hospital, Boston, Massachusetts; ^4^CDC COVID-19 Response Team; ^5^Harvard Medical School, Boston, Massachusetts; ^6^ARCHANGELS, Boston, Massachusetts; ^7^WHOOP, Inc., Boston, Massachusetts; ^8^University of Melbourne, Melbourne, Australia.

Early during the COVID-19 pandemic, nearly two thirds of unpaid caregivers of adults reported adverse mental or behavioral health symptoms, compared with approximately one third of noncaregivers^†^ (*1*). In addition, 27% of parents of children aged <18 years reported that their mental health had worsened during the pandemic (*2*). To examine mental health during the COVID-19 pandemic among U.S. adults on the basis of their classification as having a parenting role (i.e., unpaid persons caring for children and adolescents aged <18 years, referred to as children in this report) or being an unpaid caregiver of adults (i.e., persons caring for adults aged ≥18 years),^§^ CDC analyzed data from cross-sectional surveys that were administered during December 2020 and February–March 2021 for The COVID-19 Outbreak Public Evaluation (COPE) Initiative.^¶^ Respondents were categorized as parents only, caregivers of adults only, parents-caregivers (persons in both roles), or nonparents/noncaregivers (persons in neither role). Adjusted odds ratios (aORs) for any adverse mental health symptoms, particularly suicidal ideation, were higher among all respondents who were parents, caregivers of adults, or both compared with respondents who were nonparents/noncaregivers and were highest among persons in both roles (parents-caregivers) (any adverse mental health symptoms: aOR = 5.1, 95% confidence interval [CI] = 4.1–6.2; serious suicidal ideation: aOR = 8.2, 95% CI = 6.5–10.4). These findings highlight that parents and caregivers, especially those balancing roles both as parents and caregivers, experienced higher levels of adverse mental health symptoms during the COVID-19 pandemic than adults without these responsibilities. Caregivers who had someone to rely on for support had lower odds of experiencing any adverse mental health symptoms. Additional measures are needed to improve mental health among parents, caregivers, and parents-caregivers.

Among 16,384 eligible and invited unique respondents,** 10,469 (63.9%) completed English-language, Internet-based surveys administered to Qualtrics panels for The COPE Initiative during distinct intervals (December 6–27, 2020, and February 16⁠–March 8, 2021). The nonprobability demographic quota sample was weighted to closely align with the distribution of the U.S. population by sex, age, and race/ethnicity.^††^ Data for explanatory and outcome variables were obtained from 10,444 (99.8%) respondents. Respondents described their parenting and caregiving roles, completed screening instruments for symptoms of anxiety and depression^§§^ and COVID-19 trauma- and stressor-related disorders (TSRDs),^¶¶^ and reported whether during the past 30 days they had wished they were dead or could go to sleep and not wake up (passive suicidal ideation) or had seriously considered trying to kill themselves (serious suicidal ideation).***

Respondents were grouped based on their roles as 1) only parents of children aged <18 years (parents only), 2) only caregivers of adults aged ≥18 years (caregivers only), 3) having both roles (parents-caregivers), or 4) having neither role (nonparents/noncaregivers). Multivariable weighted logistic regression was used to estimate aORs by group for symptoms of anxiety or depression or COVID-19 TSRDs, passive suicidal ideation, serious suicidal ideation, or any of these symptoms. Covariates included gender, age, race/ethnicity, sexual orientation, disability status,^†††^ education, U.S. Census region^§§§^ and urbanicity^¶¶¶^ of residence, employment characteristics,**** and survey wave. Models also estimated aORs for adverse mental health symptoms by the following reasons for providing care for adults: 1) age-related health decline, 2) cognitive impairments (e.g., Alzheimer disease), 3) chronic medical conditions (e.g., cancer), 4) acute medical conditions (e.g., recovery after surgery), 5) mental health or substance use conditions, 6) active COVID-19 illness, 7) risk for severe COVID-19–associated illness, or 8) other. Additional models among all caregivers of adults, which also adjusted for parenting, duration of caregiving, hours of caregiving per week, and person receiving care, were used to estimate aORs by level of agreement with statements about caregiving-related financial strain, family strife, preparedness, support, confidence, personal freedom, positive feelings, and resentment.^††††^ Variance inflation factors for all variables with aOR estimates were less than six, indicating acceptable multicollinearity.^§§§§^

Participants provided informed electronic consent. Two-sided p values <0.05 were considered statistically significant. Rounded, weighted values are reported. Analyses were conducted using Python (version 3.7.8; Python Software Foundation) and R (version 4.0.2; R Foundation) using the R survey package (version 3.29; R Foundation). The Monash University Human Research Ethics Committee reviewed and approved the study. This activity was also reviewed by CDC and was conducted consistent with applicable federal law and CDC policy.^¶¶¶¶^

Overall, 42.5% of the 10,444 U.S. adult respondents identified as parents of children, caregivers of adults, or both, including 8.4% as parents only, 11.2% as caregivers only, and 22.9% as parents-caregivers (Table 1). Among all respondents who were parents, caregivers, or parents-caregivers, 45.0% were women and 50.2% were aged 25–44 years. The distribution by race/ethnicity was similar to those of the overall sample and the U.S. adult population. A total of 71.4% of parents or caregivers reported paid employment in addition to their parenting or unpaid caregiving roles.

**TABLE 1:** Demographic characteristics of respondents, by parent/caregiver role — The COVID-19 Outbreak Public Evaluation Initiative, United States, December 2020 and February–March 2021

Characteristic	Weighted no. (%)*					
	Total	Nonparents/ Noncaregivers	Parents only, caregivers of adults only, and parents-caregivers^†^	Parents only	Caregivers of adults only	Parents-caregivers
**Total**	**10,444 (100)**	**6,008 (57.5)**	**4,436 (42.5)**	**875 (8.4)**	**1,170 (11.2)**	**2,391 (22.9)**
**Gender^§^**						
Female	**5,138 (49.2)**	3,144 (52.3)	1,995 (45.0)	510 (58.2)	611 (52.2)	874 (36.6)
Male	**5,227 (50.1)**	2,827 (47.1)	2,400 (54.1)	360 (41.2)	552 (47.2)	1,487 (62.2)
Transgender	**58 (0.6)**	26 (0.4)	32 (0.7)	—	—	26 (1.1)
**Age group, yrs**						
18–24	**1,248 (11.9)**	549 (9.1)	699 (15.7)	91 (10.5)	145 (12.4)	462 (19.3)
25–44	**3,605 (34.5)**	1,377 (22.9)	2,227 (50.2)	426 (48.6)	393 (33.6)	1,409 (58.9)
45–64	**3,419 (32.7)**	2,293 (38.2)	1,126 (25.4)	266 (30.4)	427 (36.5)	433 (18.1)
≥65	**2,172 (20.8)**	1,789 (29.8)	384 (8.6)	92 (10.5)	205 (17.5)	87 (3.7)
**Race/Ethnicity**						
White, non-Hispanic	**6,297 (60.3)**	3,660 (60.9)	2,637 (59.5)	550 (62.9)	711 (60.7)	1,376 (57.6)
Black, non-Hispanic	**1,297 (12.4)**	766 (12.7)	531 (12.0)	103 (11.8)	135 (11.5)	293 (12.2)
Asian, non-Hispanic	**589 (5.6)**	408 (6.8)	181 (4.1)	43 (4.9)	67 (5.7)	71 (3.0)
Other, multiple races, non-Hispanic^¶^	**382 (3.7)**	220 (3.7)	162 (3.6)	36 (4.2)	61 (5.2)	64 (2.7)
Hispanic, any race	**1,880 (18.0)**	955 (15.9)	925 (20.9)	142 (16.3)	196 (16.8)	587 (24.5)
**Employment status**						
Employed	**5813 (55.7)**	2,645 (44.0)	3,167 (71.4)	518 (59.2)	654 (55.9)	1,995 (83.4)
≤40 hrs, <20% remote	**1,500 (14.4)**	970 (16.1)	531 (12.0)	153 (17.5)	179 (15.3)	199 (8.3)
≤40 hrs, 20%–80% remote	**1,209 (11.6)**	448 (7.5)	761 (17.2)	102 (11.7)	144 (12.3)	515 (21.5)
≤40 hrs, >80% remote	**877 (8.4)**	490 (8.2)	387 (8.7)	76 (8.7)	82 (7.0)	228 (9.5)
>40 hrs, <20% remote	**568 (5.4)**	341 (5.7)	227 (5.1)	66 (7.6)	79 (6.8)	81 (3.4)
>40 hrs, 20%–80% remote	**1,120 (10.7)**	224 (3.7)	896 (20.2)	80 (9.1)	117 (10.0)	699 (29.2)
>40 hrs, >80% remote	**539 (5.2)**	172 (2.9)	366 (8.3)	41 (4.7)	53 (4.6)	272 (11.4)
Unemployed	**1,791 (17.2)**	1,160 (19.3)	632 (14.2)	208 (23.8)	215 (18.4)	208 (8.7)
Retired	**2,517 (24.1)**	2,010 (33.5)	508 (11.4)	124 (14.2)	265 (22.7)	119 (5.0)
Student	**322 (3.1)**	193 (3.2)	129 (2.9)	24 (2.8)	36 (3.0)	69 (2.9)
**Duration in parenting/caregiving role**						
<3 mos	**—**	—	993 (22.4)	183 (20.9)	357 (30.5)	454 (19.0)
4–12 mos	**—**	—	1,368 (30.8)	180 (20.5)	264 (22.6)	924 (38.6)
>1 yr	**—**	—	2,075 (46.8)	513 (58.6)	549 (46.9)	1,013 (42.4)
**Parenting, hrs/wk**						
<10	**—**	—	—	145 (16.5)	—	261 (10.9)
10–20	**—**	—	—	207 (23.7)	—	377 (15.8)
21–40	**—**	—	—	211 (24.1)	—	570 (23.8)
41–60	**—**	—	—	92 (10.5)	—	374 (15.7)
>60	**—**	—	—	220 (25.2)	—	808 (33.8)
**Adult caregiving, hrs/wk**						
<10	**—**	—	—	—	317 (27.1)	239 (10.0)
10–20	**—**	—	—	—	363 (31.0)	457 (19.1)
21–40	**—**	—	—	—	229 (19.6)	606 (25.4)
41–60	**—**	—	—	—	80 (6.8)	352 (14.7)
>60	**—**	—	—	—	182 (15.6)	737 (30.8)
Reason for providing care for adults**						
Age-related health decline	**—**	—	—	—	477 (40.8)	587 (24.5)
Cognitive impairments	**—**	—	—	—	188 (16.1)	339 (14.2)
Chronic health condition	**—**	—	—	—	303 (25.9)	662 (27.7)
Acute health condition	**—**	—	—	—	118 (10.1)	405 (16.9)
Mental health or substance use condition	**—**	—	—	—	162 (13.9)	573 (24.0)
Active case of COVID-19	**—**	—	—	—	96 (8.2)	659 (27.5)
Risk for severe COVID-19	**—**	—	—	—	190 (16.3)	637 (26.6)
Other	**—**	—	—	—	165 (14.1)	155 (6.5)

* Weighted numbers and percentages might not sum to expected values because of rounding.

^†^ Parents and unpaid caregivers of adults were self-identified. Parents were defined as persons who had provided unpaid care to relatives or friends aged <18 years to help them take care of themselves at any time in the last 3 months. Unpaid caregivers of adults were defined as persons who had provided unpaid care to relatives or friends aged ≥18 years to help them take care of themselves at any time in the last 3 months. Respondents answered questions about parenting and caregiving separately. Respondents were categorized as parents only, caregivers (of adults) only, parents-caregivers (persons in both roles), or nonparents/noncaregivers. Whether adults in parenting roles were biologic or legal parents or guardians of the children for whom they were providing care is not known, nor is it known whether adults were legal dependents of their caregivers. This column includes all parents, caregivers of adults, and parents-caregivers listed in the next three columns. Weighted numbers and percentages might not sum to expected values because of rounding. Unweighted numbers and percentages for key demographic variables were as follows: survey wave (December 2020: 5,188 [49.7%]; February–March 2021: 5,256 [50.3%]), gender (female: 5,429 [52.0%]; male: 4,958 [47.5%]; transgender: 35 [0.3%]; none of these: 22 [0.2%]), age group (18–24 years: 867 [8.3%]; 25–44 years: 3,681 [35.2%]; 45–64 years: 2,994 [28.7%]; ≥65 years: 2,902 [27.8%]), and race/ethnicity (non-Hispanic White: 7,737 [74.1%]; non-Hispanic Black: 1,058 [10.1%]; non-Hispanic Asian: 529 [5.1%]; non-Hispanic other or multiple races: 353 [3.4%]; Hispanic or Latino: any race or races, 767 [7.3%]).

^§^ Respondents who chose “none of these” are not shown because of small numbers (total respondents: weighted n = 20, caregivers: weighted n = 10). Cells with counts <10 are not shown for privacy reasons.

^¶^ Includes respondents who identified as non-Hispanic and as more than one race or as American Indian or Alaska Native, Native Hawaiian or Other Pacific Islander, or all other races.

** Caregivers of adults could select multiple answers.

Approximately 70% of all caregivers (parents only, caregivers of adults only, or those with both roles) reported adverse mental health symptoms, including symptoms of anxiety or depression (55.3%), COVID-19 TSRDs (53.8%), or passive (39.3%) or serious (32.2%) suicidal ideation (Table 2). Among 2,391 parents-caregivers, approximately 85% experienced one or more adverse mental health symptoms, and approximately 50% reported past-month serious suicidal ideation. Parenting and caregiving were significantly positively associated with each adverse mental health symptom compared with being a nonparent/noncaregiver (for one or more symptoms, parents only: aOR = 1.5; caregivers only: aOR = 1.8; parents-caregivers: aOR = 5.1) and was particularly high for serious suicidal ideation among parents-caregivers compared with nonparents/noncaregivers (aOR = 8.2). Among respondents providing care for an adult for a given health condition compared with those not providing care for that condition, the highest aORs for adverse mental health symptoms were observed for caregivers of adults with mental health or substance use conditions (e.g., one or more symptoms: aOR = 5.0), adults with an active case of COVID-19 (aOR = 4.4), or adults at risk for severe COVID-19 (aOR = 3.9) (Table 2). Higher aORs for all adverse mental health symptoms were also observed for caregivers who were caring for adults with acute health conditions, chronic health conditions, cognitive impairments, and age-related health decline.

**TABLE 2:** Prevalence of and adjusted odds ratios for adverse mental health symptoms, by parent/caregiver role and reason for providing care for adults — The COVID-19 Outbreak Public Evaluation Initiative, United States, December 2020 and February–March 2021

Caregiver role and reason for providing care	Symptoms											
	Total		Anxiety or depression*		COVID-19 TSRD^†^		Past-month passive suicidal ideation^§^		Past-month serious suicidal ideation^§^		Any of these symptoms	
**Total, no. (%) (95% CI)^¶^**	**10,444 (100)**	**—**	**3,780 (36.2)**	**(35.1–37.3)**	**3,596 (34.4)**	**(33.3–35.5)**	**2,321 (22.2)**	**(21.2–23.2)**	**1,697 (16.2)**	**(15.4–17.1)**	**5,001 (47.9)**	**(46.8–49.0)**
Parent role/caregiver role,** no. (%) (95% CI)^¶^												
Nonparent/ Noncaregiver	**6,008 (57.5)**	**(56.4–58.7)**	1,327 (22.1)	(20.8–23.4)	1,209 (20.1)	(18.8–21.4)	580 (9.6)	(8.7–10.7)	269 (4.5)	(3.8–5.3)	1,925 (32.0)	(30.6–33.5)
Parent only, caregiver of adults only, or parent-caregiver	**4,436 (42.5)**	**(41.3–43.6)**	2,453 (55.3)	(53.4–57.2)	2,387 (53.8)	(51.9–55.7)	1,741 (39.3)	(37.4–41.1)	1,428 (32.2)	(30.4–34.0)	3,076 (69.3)	(67.6–71.0)
Parent only	**875 (8.4)**	**(7.8–9.0)**	315 (35.9)	(32.2–39.8)	304 (34.8)	(31.0–38.7)	162 (18.5)	(15.2–22.2)	79 (9.0)	(6.9–11.4)	443 (50.6)	(46.6–54.6)
Caregiver of adults only	**1,170 (11.2)**	**(10.4–12.0)**	454 (38.8)	(35.2–42.5)	425 (36.3)	(32.8–40.0)	187 (16.0)	(13.5–18.7)	118 (10.1)	(8.1–12.4)	591 (50.5)	(46.8–54.2)
Parent-caregiver	**2,391 (22.9)**	**(21.9–23.9)**	1,685 (70.5)	(67.9–72.9)	1,658 (69.3)	(66.8–71.8)	1,392 (58.2)	(55.6–60.9)	1,232 (51.5)	(48.8–54.2)	2,043 (85.4)	(83.5–87.2)
Parent role/caregiver role,** aOR (95% CI)^††^												
Parent only	**—**	**—**	1.4 (1.1–1.7)		1.5 (1.2–1.9)		1.5 (1.2–2.0)		1.6 (1.1–2.2)		1.5 (1.2–1.8)	
Adult caregiver only	**—**	**—**	1.9 (1.6–2.3)		1.8 (1.5–2.2)		1.3 (1.0–1.7)		1.7 (1.2–2.3)		1.8 (1.5–2.1)	
Parent-caregiver	**—**	**—**	3.7 (3.1–4.5)		3.6 (3.1–4.3)		5.8 (4.8–7.1)		8.2 (6.5–10.4)		5.1 (4.1–6.2)	
**Reason for care for adults, aOR (95% CI)^§§^**												
Age-related health decline	**—**	**—**	1.8 (1.4–2.3)		1.8 (1.5–2.3)		1.4 (1.1–1.8)		1.8 (1.3–2.3)		1.9 (1.5–2.4)	
Cognitive challenges	**—**	**—**	2.0 (1.5–2.7)		2.0 (1.5–2.6)		2.3 (1.7–3.0)		3.1 (2.2–4.4)		2.2 (1.7–2.8)	
Chronic health condition	**—**	**—**	2.8 (2.2–3.5)		1.9 (1.6–2.4)		2.8 (2.2–3.5)		3.3 (2.5–4.3)		2.3 (1.8–2.9)	
Acute health condition	**—**	**—**	2.7 (1.9–3.7)		2.8 (2.1–3.7)		2.4 (1.8–3.2)		3.6 (2.6–4.9)		3.0 (2.1–4.3)	
Mental health or substance use condition	**—**	**—**	3.7 (2.8–5.0)		3.7 (2.8–4.8)		3.0 (2.3–3.9)		3.8 (2.9–5.0)		5.0 (3.7–6.9)	
Active case of COVID-19	**—**	**—**	3.8 (2.7–5.1)		3.1 (2.3–4.1)		4.2 (3.2–5.6)		5.5 (4.1–7.5)		4.4 (3.0–6.4)	
Risk for severe COVID-19	**—**	**—**	3.4 (2.6–4.4)		2.8 (2.2–3.6)		3.5 (2.8–4.5)		4.7 (3.6–6.1)		3.9 (3.0–5.2)	
Other	**—**	**—**	1.6 (1.1–2.5)		1.4 (1.0–1.9)		1.2 (0.8–1.9)		2.4 (1.6–3.6)		1.5 (1.0–2.2)	

**Abbreviations:** aOR = adjusted odds ratio; ASD = acute stress disorder; CI = confidence interval; CII = Caregiving Intensity Index; GAD-2 = two-item Generalized Anxiety Disorder scale; IES-6 = six-item Impact of Event scale; PHQ-2 = two-item Patient Health Questionnaire; PHQ-4 = four-item Patient Health Questionnaire; PTSD = posttraumatic stress disorder; TSRD = trauma- and stressor-related disorder.

* Symptoms of anxiety and depression were assessed via PHQ-4. Those who scored ≥3 out of 6 on the GAD-2 or PHQ-2 subscales were considered symptomatic for anxiety or depression symptoms.

^†^ Disorders classified as TSRDs in the *Diagnostic and Statistical Manual of Mental Disorders, Fifth Edition,* include PTSD, ASD, and adjustment disorders, among others. Symptoms of a TSRD attributed to the COVID-19 pandemic were assessed via IES-6 to screen for overlapping symptoms of PTSD, ASD, and adjustment disorders. The COVID-19 pandemic was specified as the traumatic exposure to record peritraumatic and posttraumatic symptoms associated with the range of stressors introduced by the COVID-19 pandemic. Those who scored ≥1.75 out of 4 were considered symptomatic.

^§^ Passive suicidal ideation was assessed using an item from the Columbia-Suicide Severity Rating Scale adapted to refer to the past 30 days: “At any time in the past 30 days, have you wished you were dead or wished you could go to sleep and not wake up?” Serious suicidal ideation was assessed using an item from the National Survey on Drug Use and Health adapted to refer to the past 30 days: “At any time in the past 30 days, did you seriously think about trying to kill yourself?”

^¶^ Weighted numbers and percentages might not sum to expected values because of rounding.

** Parents and unpaid caregivers of adults were self-identified. For this analysis, parents were defined as persons who had provided unpaid care to relatives or friends aged <18 years to help them take care of themselves at any time in the last 3 months. Unpaid caregivers of adults were defined as persons who had provided unpaid care to relatives or friends aged ≥18 years to help them take care of themselves at any time in the last 3 months. Respondents answered questions about these two roles separately. Respondents were categorized as parents only, caregivers of adults only, parents-caregivers (persons in both roles), or nonparents/noncaregivers. Whether adults in parenting roles were biologic or legal parents or guardians of the children for whom they were providing care is not known, nor is it known whether adults were legal dependents of their caregivers.

^††^ Referent: nonparent/noncaregiver. Weighted multivariable logistic regression models were used to estimate aORs for each adverse mental health symptom, with survey wave, gender, age group, race/ethnicity, sexual orientation, disability status, education attainment, region, urbanicity, and employment (work hours per week and remote work percentage) as covariates. P values <0.05 were considered statistically significant. Models with all unpaid caregiver statuses included 10,017 respondents because persons who answered “prefer not to say” for sexual orientation or disability status and those who reported invalid zip codes were excluded. Models with unpaid caregivers of adults included 3,155 respondents; respondents were excluded for the same reasons.

^§§^ Referent: not providing care to an adult for this reason. This referent group includes all adults not providing care for the listed reason, including those who were nonparents/noncaregivers, parents only, and caregivers of adults who were providing care for different reasons.

Among all caregivers of adults (adult caregivers only and parents-caregivers), those who agreed with the statements that they had experienced caregiving-related family disagreements or resented their caregiving responsibilities had approximately three times the odds for any adverse mental health symptoms (Figure) compared with those who disagreed with these statements. Similarly, aORs for any adverse mental health symptoms were approximately twice as high for caregivers who agreed that they felt underprepared as a caregiver, did not have the personal freedom they desired, or had to decrease living expenses to help pay for things, compared with caregivers who did not agree with these statements. Conversely, persons who had someone to rely on for support had lower odds of experiencing any adverse mental health symptoms.

**FIGURE:**
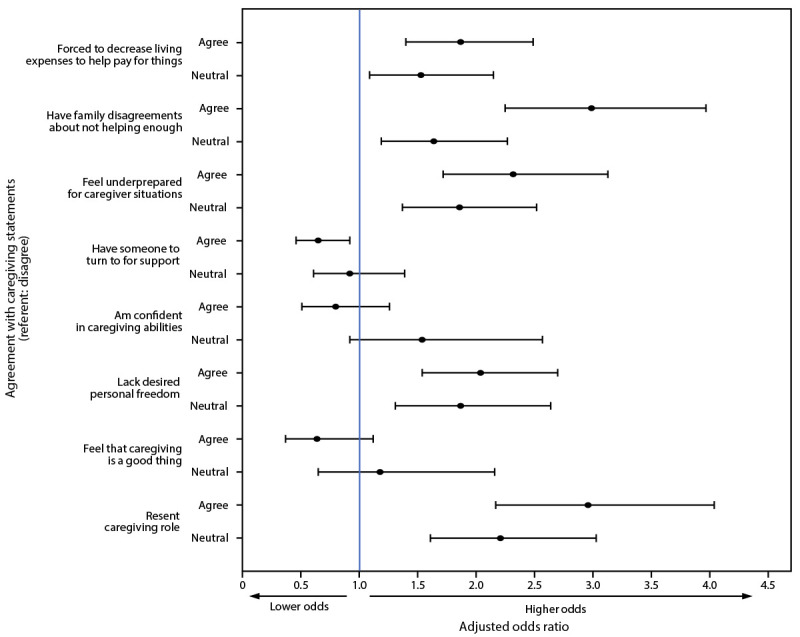
Factors* associated^†^ with adverse mental health symptoms^§^ among unpaid caregivers of adults and parents-caregivers^¶^ — The COVID-19 Outbreak Public Evaluation Initiative, United States, December 2020 and February–March 2021 **Abbreviations:** ASD = acute stress disorder; CII = Caregiving Intensity Index; GAD-2 = two-item Generalized Anxiety Disorder scale; IES-6 = six-item Impact of Event scale; PHQ-2 = two-item Patient Health Questionnaire; PHQ-4 = four-item Patient Health Questionnaire; PTSD = posttraumatic stress disorder; TSRD = trauma- and stressor-related disorder. * Caregiving statements were taken from the ARCHANGELS short-form CII, a copyrighted instrument available for use only with permission. ^†^ Adjusted odds ratios, with 95% confidence intervals indicated by error bars, were estimated using weighted multivariable logistic regression models. The primary model is adjusted for survey wave, gender, age group, race/ethnicity, sexual orientation, disability status, education attainment, region, urbanicity, and employment (including work hours per week and remote work percentage), parental status (i.e., whether caregivers were parents-caregivers), duration of caregiving, hours of caregiving per week, and person receiving care. Additional separate models were analyzed for each CII item that was based on perceived levels of agreement with statements regarding caregiving-related financial strain, family strife, preparedness, support, confidence, personal freedom, positive feelings, and resentment. ^§^ The presence of one or more of the following was considered an adverse mental health symptom: anxiety symptoms, depression symptoms, COVID-19 TSRD symptoms, passive suicidal ideation, or having seriously considered suicide in the past 30 days. Symptoms of anxiety and depression were assessed via PHQ-4. Those who scored ≥3 out of 6 on the GAD-2 and PHQ-2 subscales were considered symptomatic for these respective conditions. Disorders classified as TSRDs in the *Diagnostic and Statistical Manual of Mental Disorders, Fifth Edition,* include PTSD, ASD, and adjustment disorders, among others. Symptoms of a TSRD attributed to the COVID-19 pandemic were assessed via IES-6 to screen for overlapping symptoms of PTSD, ASD, and adjustment disorders. The COVID-19 pandemic was specified as the traumatic exposure to record peritraumatic and posttraumatic symptoms associated with the range of stressors introduced by the COVID-19 pandemic. Those who scored ≥1.75 out of 4 were considered symptomatic. Passive suicidal ideation was assessed using an item from the Columbia-Suicide Severity Rating Scale adapted to refer to the past 30 days: “At any time in the past 30 days, have you wished you were dead or wished you could go to sleep and not wake up?” Serious suicidal ideation was assessed using an item from the National Survey on Drug Use and Health, adapted to refer to the past 30 days: “At any time in the past 30 days, did you seriously think about trying to kill yourself?” ^¶^ Parents and unpaid caregivers of adults were self-identified. Unpaid caregivers of adults were defined as persons who had provided unpaid care to relatives or friends aged ≥18 years to help them take care of themselves at any time in the last 3 months. Parents were defined as persons who had provided unpaid care to relatives or friends aged <18 years to help them take care of themselves at any time in the last 3 months. Parents-caregivers had both roles. All unpaid caregivers of adults were included in this analysis, including caregivers of adults only (i.e., not parents) and parents-caregivers.

## Discussion

Approximately 40% of U.S. adults surveyed in late 2020 or early 2021 reported having parenting responsibilities, adult caregiving responsibilities, or both. Overall, 70% of all caregivers (parents only, caregivers of adults only, or those with both roles) reported recent adverse mental health symptoms, including symptoms of anxiety or depression, COVID-19 TSRDs, or suicidal ideation. Of particular concern, 85% of respondents with both parenting responsibilities and adult caregiving responsibilities experienced adverse mental health symptoms, and approximately 50% reported past-month serious suicidal ideation, with eight times the odds of serious suicidal ideation compared with nonparents/noncaregivers.

Caregivers of adults with mental health or substance use conditions, adults currently ill with COVID-19, or adults at risk for severe COVID-19 reported more adverse mental health symptoms than did caregivers of adults with other conditions, highlighting the need for education and support for caregivers in these roles. Social factors, such as financial strain, feeling a lack of preparedness for or resentment of caregiving, a lack of freedom, and family conflict were also associated with adverse mental health. The lower odds of having any adverse mental health symptoms based on the perception of having a person to rely on for support is encouraging. Because employment and caregiving responsibilities might limit the time available to seek help, telehealth and Internet-based interventions (*3*) might improve caregiver mental health; however, Internet access might be limited for some populations, particularly those with lower incomes. In addition, adult day services centers might benefit the mental health of caregivers and of those for whom they are providing care (*4*).***** Finally, suicide prevention^†††††^ and mental health disaster support services^§§§§§^^,^^¶¶¶¶¶^^,^****** are needed for parents and caregivers.

During the pandemic, parents and caregivers have had worse mental health than adults without parenting and caregiving responsibilities (*5*). Managing mental health might be especially challenging for parents balancing employment and remote education; virtual instruction during the COVID-19 pandemic has presented risks for mental health both among children and parents (*6*). For caregivers of adults, these findings reinforce prepandemic data on poor mental health among caregivers (*7*). The results also support AmeriSpeak Omnibus survey findings that during the COVID-19 pandemic, caregivers had substantial concerns about their own mental health and the health and well-being of their care recipients, were worried about their finances, and needed respite from caregiving (*8*). Adverse mental health consequences for persons in both roles (i.e., parents-caregivers) support an urgent need to tailor public health efforts to this population. Together, these results suggest that parents and caregivers might benefit from tailored mental health services. For caregivers, and especially persons with dual responsibilities of parenting while also caring for adults, increasing access to, awareness of, and use of support groups and respite services^††††††^ might help to alleviate the caregiving workload^§§§§§§^ (*9*).

The findings in this report are subject to at least six limitations. First, this study did not fully characterize parenting roles (e.g., age and number of children, whether children had chronic health conditions, and whether children were in virtual rather than in-person school). Whether the mental health of adults differs based on these factors could be explored. Second, diagnostic evaluations for anxiety and depression were not conducted; however, clinically validated instruments were used to measure symptoms of anxiety and depression. Third, responses might be subject to social desirability bias, particularly regarding negative feelings about caregiving roles, which might be underreported. Fourth, without prepandemic mental health data in this sample, whether adverse mental health symptoms were caused by or worsened by the pandemic is unknown. However, caregivers of adults had higher odds of new adverse mental or behavioral health symptoms during the pandemic than did noncaregivers (*1*). Fifth, the survey did not assess support systems for parents or caregivers (e.g., child care or support from family members), which could have affected the intensity of their caregiving roles. Finally, because the surveys were English-language only and quota sampling and survey weighting might not have eliminated inherent biases in Internet-based survey samples,^¶¶¶¶¶¶^ this sample might not fully represent the U.S. population, particularly regarding English-language fluency and Internet access. This might partially account for the finding that more parents, caregivers, or parents-caregivers were male. However, previous studies have estimated that up to 47% of caregivers are male. Furthermore, the infrequency of assessments of both parental and caregiving roles makes comparing these estimates difficult.******* The prevalence and trajectories of anxiety and depression symptoms were consistent with results from the Household Pulse Survey^†††††††^ (*10*), and robust associations between parenting and caregiving roles and adverse mental health symptoms in the large, demographically diverse COPE Initiative sample merit additional research.

Caregivers, particularly persons with both parenting and adult caregiving responsibilities, will continue to face mental health challenges, and the need for caregivers is projected to increase as the U.S. population ages.^§§§§§§§^ Additional research can assess differences in coping and help-seeking behaviors among parents and caregivers to further guide tailored support and services to meet their needs during and after the COVID-19 pandemic.

**Summary**

**What is already known about this topic?**

Parents of children aged <18 years and unpaid caregivers of adults have had mental health challenges before and during the COVID-19 pandemic.

**What is added by this report?**

Among 10,444 U.S. adults surveyed during December 6–27, 2020, and February 16⁠–March 8, 2021, parents, unpaid caregivers of adults, and parents-caregivers (persons in both roles) had significantly worse mental health than adults not in these roles, including five times the odds of any adverse mental health symptoms (parents-caregivers). Persons who had someone to rely on for support had lower odds of experiencing any adverse mental health symptoms.

**What are the implications for public health practice?**

Parents and unpaid caregivers of adults, and particularly those in both roles, might benefit from mental health support and services tailored to their roles.

## Footnotes

* These authors contributed equally to this report.

†
https://www.medrxiv.org/content/10.1101/2021.02.02.21251042v1

§ Parents and unpaid caregivers of adults were self-identified. Parents were defined as persons who had provided unpaid care to relatives or friends aged <18 years to help them take care of themselves at any time during the last 3 months. Unpaid caregivers of adults were defined as persons who had provided unpaid care to relatives or friends aged ≥18 years to help them take care of themselves at any time during the last 3 months. Respondents answered questions about these two roles separately. Respondents were categorized as parents only, caregivers of adults only, parents-caregivers (persons in both roles), or nonparents/noncaregivers. Whether adults who reported they were in parenting roles were biologic or legal parents or guardians of the children for whom they were providing care is not known, nor is it known whether adults were legal dependents of their caregivers.

¶ The COPE Initiative (https://www.thecopeinitiative.org/) is designed to assess public attitudes, behaviors, and beliefs related to the COVID-19 pandemic and to evaluate the mental and physical health consequences of the pandemic. The COPE Initiative surveys included in this analysis were administered by Qualtrics, LLC (https://www.qualtrics.com), a commercial survey company with a network of participant pools comprising hundreds of suppliers and with varying recruitment methodologies that include digital advertisements and promotions, word-of-mouth and membership referrals, social networks, television and radio advertisements, and offline mail-based approaches.

** Eligibility to complete surveys was determined after electronic contact with potential participants who met criteria of age ≥18 years and U.S. residence. Age and residence were assessed using screening questions without indication of eligibility criteria before survey commencement. Country-specific geolocation verification via IP address mapping was used to ensure respondents were in the United States. Qualtrics, LLC, conducted data quality screening including algorithmic and keystroke analysis for attention patterns, click-through behavior, duplicate responses, machine responses, and inattentiveness.

†† Additional information on quota sampling, a nonprobabilistic sampling method, is available at https://www.qualtrics.com/experience-management/research/sampling-methods/. Demographic quotas were set for sex, age, race, and ethnicity using questions and national U.S. adult population estimates from the 2019 American Community Survey. After the surveys were conducted, iterative proportional fitting and weight trimming were applied to the overall sample to match 2019 American Community Survey estimates for sex, age, and combined race/ethnicity. Survey weighting was performed using the R survey package (version 3.29; R Foundation).

§§ Symptoms of anxiety and depression were assessed via the four-item Patient Health Questionnaire (PHQ-4), which refer to anxiety and depression symptoms experienced over the past 2 weeks. Those who scored ≥3 out of 6 on the Generalized Anxiety Disorder (GAD-2) or Patient Health Questionnaire (PHQ-2) subscales were considered symptomatic for these respective conditions.

¶¶ Disorders classified as TSRDs in the *Diagnostic and Statistical Manual of Mental Disorders, Fifth Edition,* include posttraumatic stress disorder (PTSD), acute stress disorder (ASD), and adjustment disorders, among others. Symptoms of a TSRD attributed to the COVID-19 pandemic were assessed via the six-item Impact of Event scale (IES-6) to screen for overlapping symptoms of PTSD, ASD, and adjustment disorders. The COVID-19 pandemic was specified as the traumatic exposure to record peritraumatic and posttraumatic symptoms associated with the range of stressors introduced by the COVID-19 pandemic. Those who scored ≥1.75 out of 4 were considered symptomatic.

*** For questions related to suicidal ideation, participants were informed that responses were deidentified and that direct support could not be provided to those who reported substance use behavior or suicidal ideation. Regarding suicidal ideation, all respondents were provided the following: “This survey is anonymous, so we cannot provide direct support. If you would like crisis support, please contact the National Suicide Prevention Lifeline, 1-800-273-TALK (8255, or chat line) for help for yourself or for others.” Passive suicidal ideation was assessed using an item from the Columbia-Suicide Severity Rating Scale adapted to refer to the past 30 days: “At any time in the past 30 days, have you wished you were dead or wished you could go to sleep and not wake up?” Serious suicidal ideation was assessed using an item from the National Survey on Drug Use and Health adapted to refer to the past 30 days: “At any time in the past 30 days, did you seriously think about trying to kill yourself?”

††† Persons who had a disability were defined as such based on a qualifying response to either one of the two following questions: “Are you limited in any way in any activities because of physical, mental, or emotional condition?” and “Do you have any health conditions that require you to use special equipment, such as a cane, wheelchair, special bed, or special telephone?” https://www.cdc.gov/brfss/questionnaires/pdf-ques/2015-brfss-questionnaire-12-29-14.pdf

§§§
https://www2.census.gov/geo/pdfs/maps-data/maps/reference/us_regdiv.pdf

¶¶¶
https://www.hrsa.gov/rural-health/about-us/definition/datafiles.html

**** Employment characteristics included employment status (employed, unemployed, retired, or student only), weekly paid work hours, and percentage of work hours completed remotely versus on-site.

†††† Respondents rated their level of agreement to statements using a five-item Likert scale (1 = strongly disagree; 2 = disagree; 3 = neither agree nor disagree; 4 = agree; 5 = strongly agree) taken from the ARCHANGELS short-form Caregiver Intensity Index (CII), a copyrighted instrument available for use only with permission. Responses were trichotomized to disagree (1 and 2), neutral (3), or agree (4 and 5). CII was administered to all unpaid caregivers of adults.

§§§§ The maximum acceptable level of variance inflation factor cutoff was set at 10, which signals high multicollinearity (i.e., when two or more explanatory variables in a multivariable model are highly correlated). https://www.itl.nist.gov/div898/software/dataplot/refman2/auxillar/vif.htm

¶¶¶¶ 45 C.F.R. part 46, 21 C.F.R. part 56; 42 U.S.C. Sect. 241(d); 5 U.S.C. Sect. 552a; 44 U.S.C. Sect. 3501 et seq.

*****
https://www.cdc.gov/coronavirus/2019-ncov/community/adult-day-care-service-centers.html

††††† National Suicide Prevention Lifeline (https://suicidepreventionlifeline.org/) or Lifeline Crisis Chat (https://suicidepreventionlifeline.org/chat/).

§§§§§ Substance Abuse and Mental Health Services Administration National Helpline (also known as the Treatment Referral Routing Service) for persons and families facing mental disorders, substance use disorders, or both (https://www.samhsa.gov/find-help/national-helpline).

¶¶¶¶¶ Disaster Distress Helpline (https://www.samhsa.gov/disaster-preparedness).

****** Crisis Text Line (https://www.crisistextline.org/).

†††††† https://www.cdc.gov/aging/publications/features/caring-for-yourself.html; https://www.norc.org/PDFs/Maintaining%20Physical%20and%20Mental%20Well/OACCaregiverOnePager.pdf

§§§§§§
https://www.caregiving.org/resources/

¶¶¶¶¶¶
https://www.pewresearch.org/politics/methodology/collecting-survey-data/internet-surveys/

*******
https://www.apa.org/pi/about/publications/caregivers/faq/statistics

†††††††
https://www.cdc.gov/nchs/covid19/pulse/mental-health.htm

§§§§§§§
https://www.cdc.gov/aging/caregiving/index.htm
